# Estimating cardiac output based on gas exchange during veno-arterial extracorporeal membrane oxygenation in a simulation study using paediatric oxygenators

**DOI:** 10.1038/s41598-021-90747-w

**Published:** 2021-06-01

**Authors:** Kaspar Felix Bachmann, Rakesh Vasireddy, Paul Philipp Heinisch, Hansjörg Jenni, Andreas Vogt, David Berger

**Affiliations:** 1 Department of Anaesthesiology and Pain Medicine, Inselspital, Bern University Hospital, University of Bern, Bern, Switzerland; 2 Department of Intensive Care Medicine, Inselspital, Bern University Hospital, University of Bern, Bern, Switzerland; 3 Department of Cardiac and Vascular Surgery, Inselspital, Bern University Hospital, University of Bern, Bern, Switzerland; 4 Department of Congenital and Pediatric Heart Surgery, German Heart Center Munich, Technische Universität München, Munich, Germany

**Keywords:** Cardiac device therapy, Experimental models of disease, Translational research, Heart failure, Biomedical engineering

## Abstract

Veno-arterial extracorporeal membrane oxygenation (VA-ECMO) therapy is a rescue strategy for severe cardiopulmonary failure. The estimation of cardiac output during VA-ECMO is challenging. A lung circuit (${\dot{\text{Q}}}$_Lung_) and an ECMO circuit (${\dot{\text{Q}}}$_ECMO_) with oxygenators for CO_2_ removal ($\mathop {\text{V}}\limits^{.}$CO_2_) and O_2_ uptake ($\mathop {\text{V}}\limits^{.}$O_2_) simulated the setting of VA-ECMO with varying ventilation/perfusion ($\mathop {\text{V}}\limits^{.}$/${\dot{\text{Q}}}$) ratios and shunt. A metabolic chamber with a CO_2_/N_2_ blend simulated $\mathop {\text{V}}\limits^{.}$CO_2_ and $\mathop {\text{V}}\limits^{.}$O_2_. ${\dot{\text{Q}}}$_Lung_ was estimated with a modified Fick principle: ${\dot{\text{Q}}}$_Lung_ = ${\dot{\text{Q}}}$_ECMO_ × ($\mathop {\text{V}}\limits^{.}$ CO_2_ or $\mathop {\text{V}}\limits^{.}$O_2Lung_)/($\mathop {\text{V}}\limits^{.}$CO_2_ or $\mathop {\text{V}}\limits^{.}$O_2ECMO_). A normalization procedure corrected $\mathop {\text{V}}\limits^{.}$CO_2_ values for a $\mathop {\text{V}}\limits^{.}$/${\dot{\text{Q}}}$ of 1. Method agreement was evaluated by Bland–Altman analysis. Calculated ${\dot{\text{Q}}}$_Lung_ using gaseous $\mathop {\text{V}}\limits^{.}$CO_2_ and $\mathop {\text{V}}\limits^{.}$O_2_ correlated well with measured ${\dot{\text{Q}}}$_Lung_ with a bias of 103 ml/min [− 268 to 185] ml/min; Limits of Agreement: − 306 ml/min [− 241 to − 877 ml/min] to 512 ml/min [447 to 610 ml/min], r^2^ 0.85 [0.79–0.88]). Blood measurements of $\mathop {\text{V}}\limits^{.}$CO_2_ showed an increased bias (− 260 ml/min [− 1503 to 982] ml/min), clinically not applicable. Shunt and $\mathop {\text{V}}\limits^{.}$/${\dot{\text{Q}}}$ mismatch decreased the agreement of methods significantly. This in-vitro simulation shows that $\mathop {\text{V}}\limits^{.}$CO_2_ and $\mathop {\text{V}}\limits^{.}$O_2_ in steady-state conditions allow for clinically applicable calculations of ${\dot{\text{Q}}}$_Lung_ during VA-ECMO therapy.

## Introduction

Shock states and lung failure are the most common reasons for admission to an intensive care unit. Both carry considerable morbidity and mortality and are amongst the leading causes of death in the developed world. Extracorporeal membrane oxygenation (ECMO) has gained widespread interest as a rescue therapy for severe pulmonary or circulatory failure and its use grows exponentially^1^.

ECMO provides an extracorporeal support for functions of the lung and heart. It may serve as a bridge to recovery or long-term mechanical assist devices and transplantation. In a parallel connection to the patient’s own circulation, veno-arterial ECMO drains venous blood from the patient into an extracorporeal membrane lung, where carbon dioxide is removed and hemoglobin in the red blood cells is oxygenated. The arterialized (oxygenated and decarboxylized) blood is pumped back into the patient’s arterial system. It is a concept similar to the cardiopulmonary bypass in heart surgery (“heart–lung-machine”), where patients undergo extracorporeal circulation on a daily basis. ECMO however is suitable for long-term support^2^.

ECMO treatment is technically demanding. In a recent review, refinement of patient inclusion criteria, optimization of additional treatment strategies and weaning strategies were considered research fields of major importance for the ongoing improvement for patients on ECMO treatment^3^. The physiology of gas exchange and blood flow with two competing systems, the ECMO and the patient’s own heart and lung in parallel connection, is incompletely understood^4^. The goal of the treatment is to maintain tissue perfusion and gas exchange in order to gain time for recovery of native cardiac output (i.e. blood flow through the lungs). The assessment of cardiac output under ongoing extracorporeal treatment and especially during weaning, i.e. the stepwise liberation from the extracorporeal support, is difficult and mainly based on expert opinion^5,6^. Novel experimental approaches including modified thermodilution exist^7^, but are not validated in a clinical setting. Timing and strategy for weaning ECMO are complex issues^5^, whereby early weaning is linked to a favorable outcome^1,8,9^. During this weaning procedure, where $\mathop {\text{V}}\limits^{.}$/${\dot{\text{Q}}}$ mismatch is improving, we see the possibility of assessing pulmonary blood flow through gas exchange as a useful tool to support the clinician at the bedside.

Recently, we investigated the non-invasive estimation of native cardiac output during ECMO using an adaptation of the Fick principle and expiratory gas measurements^10^. By conceptually treating the ECMO circuit as a right-to-left shunt, we created a mathematical model yielding pulmonary blood flow (i.e. cardiac output) from ECMO blood flow and from measurements of carbon dioxide elimination and oxygen consumption ($\mathop {\text{V}}\limits^{.}$O_2_ and $\mathop {\text{V}}\limits^{.}$O_2_) through the membrane lung and the natural lung. As the ventilation / perfusion ratio ($\mathop {\text{V}}\limits^{.}$/${\dot{\text{Q}}}$ ratio) has a major influence on the amount of CO_2_ eliminated^11,12^, we additionally estimated a correction factor to account for the non-linearity in CO_2_ elimination^10^.

To confirm our preliminary results and model, we built an in-vitro lung/ECMO simulator by adapting an experimental setup developed at the department of Anaesthesiology and Pain Medicine, University Hospital of Bern, Switzerland^13^. In vitro models have successfully been used to simulate aspects of ECMO therapy such as flow characteristics^14^, platelet activation^15^, delivering of therapeutic enzymes^16^ or energy loss due to components of the circuit^17^.

The aim of this simulator study was to evaluate our modified Fick method under changing $\mathop {\text{V}}\limits^{.}$/${\dot{\text{Q}}}$ ratios and shunt and to evaluate the influence of these on the accuracy of the method. Furthermore, we compared the carbon dioxide contents in the blood and gas phase and their relationship to respective blood flows. According to the 3R principles for replacement, reduction, and refinement of the use of animals in experimentation, an additional simulation of our preliminary data from a small proof-of-concept study before confirming it in a larger trial will allow us to refine our techniques and define the limiting factors for the method more clearly.

## Materials and methods

**Figure 1:**
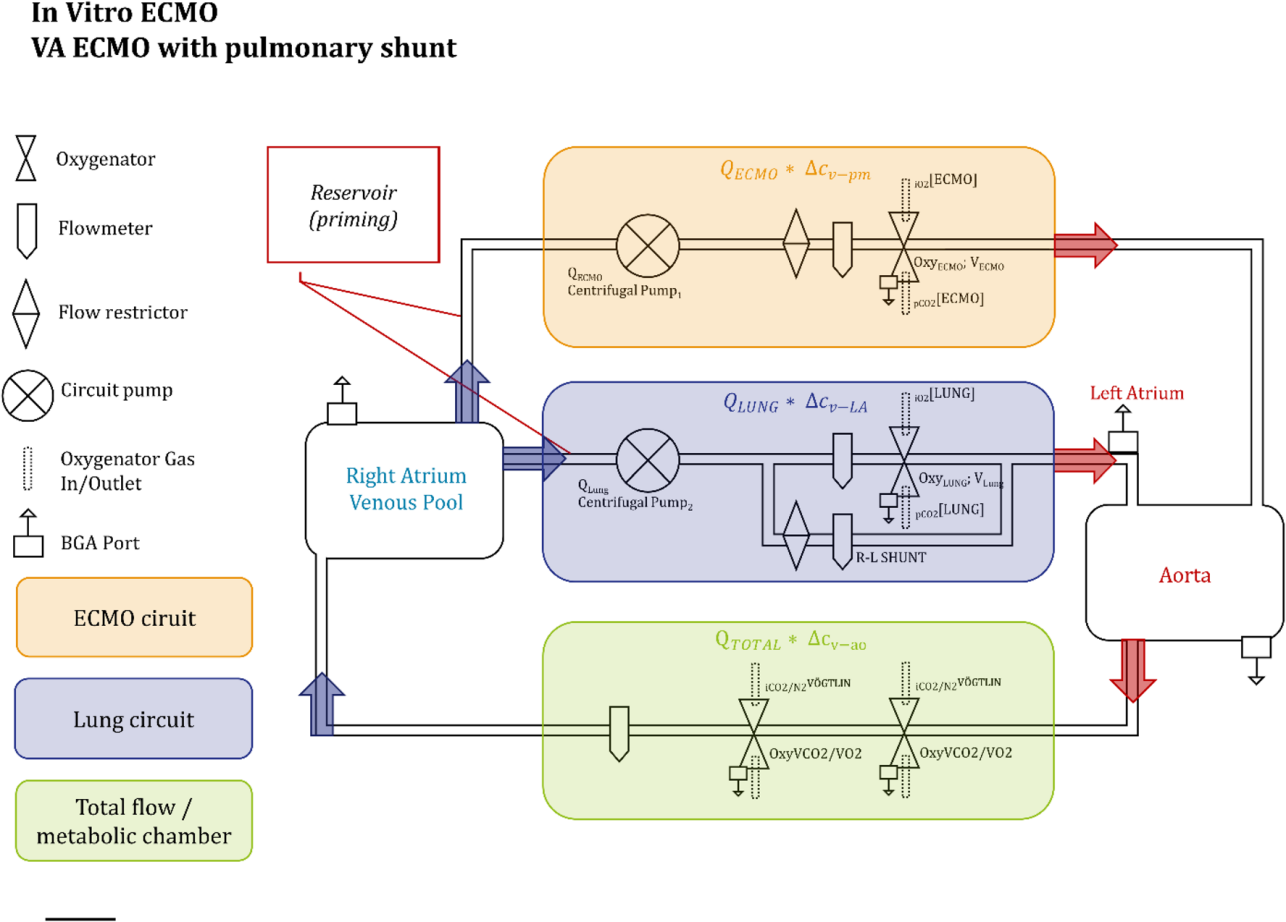
In-vitro lung/ECMO simulation. The in-vitro simulation consists of two parallel circuits (ECMO and lung) with the ability to shunt Oxy_Lung_. Blood samples could be drawn after Oxy_ECMO_ (post membrane), after Oxy_Lung_ (post lung), from the left atrium (LA), the aorta as well as the right atrium (RA).

The simulation consisted of two parallel circuits—one representing the ECMO blood flow with extracorporeal gas exchange, the other lung and heart—merged into the systemic circulation (Fig. 1). One circuit represents the human heart and the lung: It consisted of a micro-diagonal pump (DeltaStream DP-II, Medos, Stolberg, Germany), generating non-pulsatile flow, as the heart and an oxygenator (Oxy_Lung_ QUADROX-i Pediatric Oxygenators; MAQUET, Hirrlingen, Germany) as the natural lung, including a blood flow bypass around the Oxy_LUNG_ for the simulation of anatomical or functional right-to-left shunt. The second circuit, consisting of the same type of pump and oxygenators, represents the ECMO (Oxy_ECMO_). Both oxygenators were operated at a fraction of inspired oxygen of 50% throughout the experiment. These two circuits (Lung and ECMO) were merged into one mixed flow, representing the Aorta and then guided into a simulated metabolic chamber. Here, over two oxygenators (Oxy_VCO2/O2_, Terumo Capiox RX25R, Ann Arbor, MI, USA) carbon dioxide was introduced into the system and oxygen washed out with a nitrogen/carbon dioxide gas blend to ensure venous pCO_2_ values between 50 and 80 mmHg and mixed venous saturations of 70–90%. Gas flows were regulated with high precision flow control valves (Vögtlin RED-Y, Basel-Land, Switzerland). Blood was collected in a venous, air-free, reservoir bag above the functional right atrium to ensure steady perfusate supply at different blood flow rates. Blood flows between the circuits and the shunt were regulated with simple flow restrictors (adjusting nuts).

Priming volume of the system was approximately 2.2 L. It was filled with a mixture of discarded human red blood cells and lactated Ringer’s solution in a ratio of 3:1 to reach a hemoglobin value of 8–10 g/L. 50–100 mmol of sodium bicarbonate were added to reach physiological pH values between 7.3–7.4^4^. Glucose 20% was added to keep glucose level above 5 mmol/l. Boluses of 5000 I. E. Heparin were added every 2–3 h to prevent clotting. The system was heated to 36.8 °C using a temperature control system (HCV, Type 20-602, Jostra Fumedica, Muri, Switzerland).

### Measurements

Exhaust P_E_CO_2_ at the ECMO was measured using a standard side-stream capnometer (Vamos, Dräger, Lübeck, Germany) with a constant 200 ml/min side-stream flow and a measurement accuracy of ± 3.3 mmHg + 8% relative error, as specified by the manufacturer. After every experimental maneuver, blood gas samples were drawn at specified ports and analyzed with a point of care device (Cobas b 123, Roche Diagnostics, Basel, Switzerland). Blood flows were continuously measured using liquid flow meters (Levitronix, Zurich, Switzerland, Fig. 1). Sweep gas flows were set and recorded manually at the gas blenders for Oxy_Lung_ and Oxy_ECMO_ and digitally using flow control valves for Oxy$_{{\mathop {\text{V}}\limits^{.} {\text{CO}}_{2} /\mathop {\text{V}}\limits^{.} {\text{O}}_{2} }}$.

### Study protocol

#### Baseline

We aimed at a total flow of 2500 ml in the systemic circulation, measured after the metabolic chamber. At baseline, this flow was partitioned in 2000 ml/min running through the ECMO circuit and 500 ml/min running through the lung circuit. Aortal pCO_2_ at baseline was aimed at 40 mmHg corresponding to a simulated CO_2_ production of approximately 120–150 ml/min. In a clinical setting the pulmonary blood flow is unknown and establishing steady $\mathop {\text{V}}\limits^{.}$/${\dot{\text{Q}}}$ or its prediction is not possible. Therefore, lung gas flow was kept constant at 1.5 l/min (F_i_O_2_ 50%) and remained unchanged during each experimental step.

#### Step one

**Figure 2:**
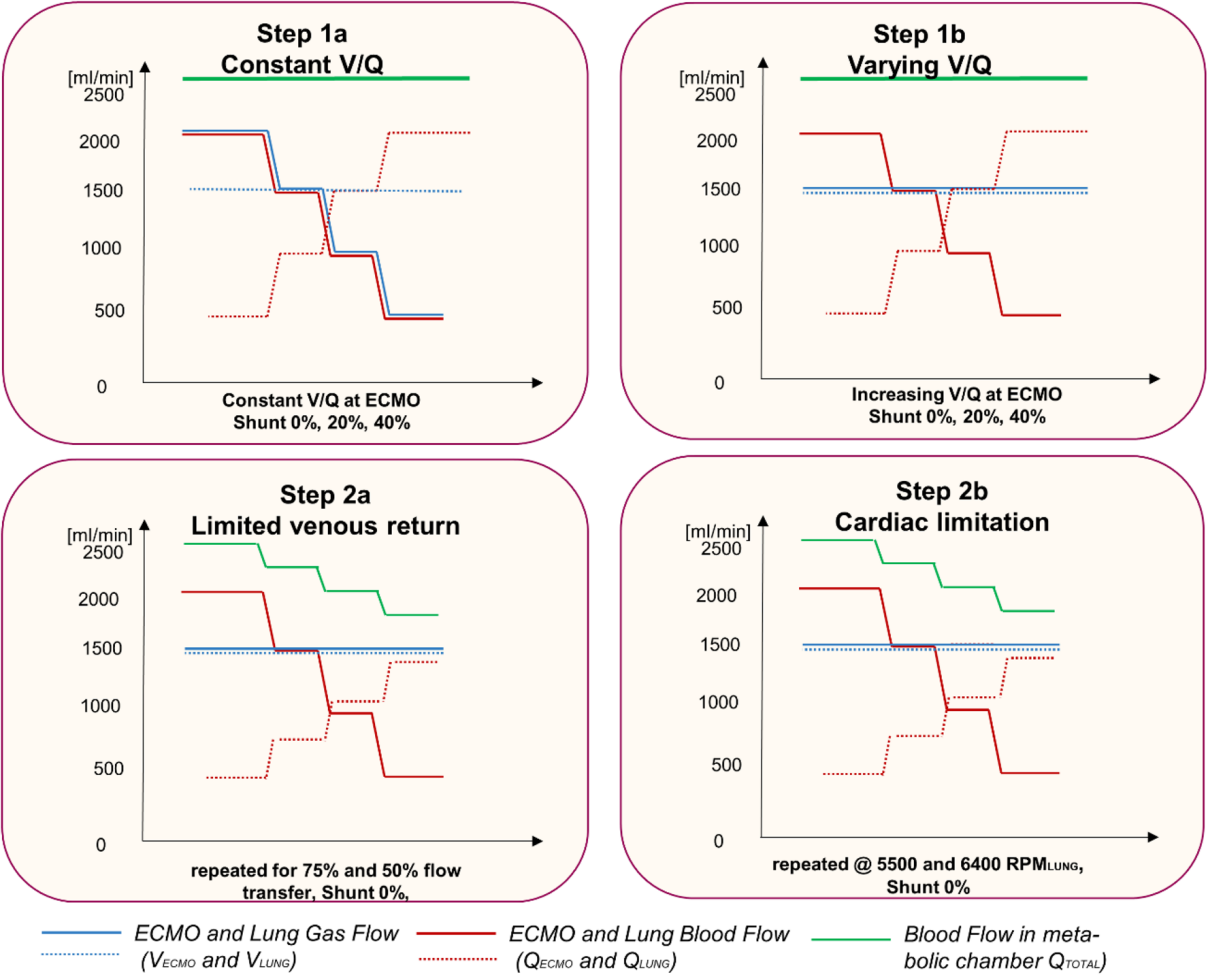
Step 1a and 1b. Experimental Manoeuvres (Step 1a, 1b, 2a, 2b). In the first part, weaning of ECMO blood flow was conducted with reductions of 500 ml and corresponding equal̇ increases of lung blood flow, summing up to a systemic blood flow of 2500 ml. The effect of $\mathop {\text{V}}\limits^{.}$/${\dot{\text{Q}}}$ was evaluated at a constant and varying $\mathop {\text{V}}\limits^{.}$/${\dot{\text{Q}}}$ ratio (Step a and b, respectively). Both manoeuvres were triplicated at 0%, 20% and 40% pulmonary shunt. Step 2a and 2b. A second part investigated the effects of limiting venous return (Step a) or limited pump function (step b). Venous return was limited by incomplete transfer of the weaned ECMO blood flow to lung blood flow (25% and 50% reduction), leading to venous pooling of blood. Limited cardiac function was simulated by a constant RPM at the lung circuit.

From this baseline, multiple weaning trials were performed by 500-ml-wise reductions of ECMO blood flow with either a constant $\mathop {\text{V}}\limits^{.}$/${\dot{\text{Q}}}$ ratio of 1 (gas flow matches blood flow; Fig. 2 Step 1a) or varying $\mathop {\text{V}}\limits^{.}$/${\dot{\text{Q}}}$ ratios of 3, 1.5, 1 and 0.75 (constant gas flow of 1.5 l/min during reduction of blood flow; Fig. 2 Step 1b) on the ECMO and consecutive increases in lung blood flow 500 ml, matching the ECMO blood flow reduction. These maneuvers were repeated for shunt fractions of 0%, and 20% and 40%.

#### Step two

In a second step—in order to investigate the situation of limited venous return or limited cardiac function—lung blood flow was not directly regulated but was the indirect result of changing the venous pool at unchanged rotations per minute (RPM; Fig. 2, Step 2a). Cardiac limitation was simulated by adjusting the RPM of the Lung pump (Fig. 2, Step 2b). For these second steps, shunt was kept constant at 0%.

## Calculations and mathematical model

### Blood flow calculations

Based on the ventilation/perfusion ($\mathop {\text{V}}\limits^{.}$/${\dot{\text{Q}}}$) concept from respiratory physiology, gas exchange of the native lung is proportional to its blood flow^18^. The Fick principle and mass balance equations (Formula A–C) allow the deduction of formula (1) ^10,19^:

$$Q_{total} = Q_{LUNG} + Q_{ECMO} \quad \left( A \right)$$

$$VCO_{2\,total} = VCO_{2\,LUNG} + VCO_{2\,ECMO} { } \quad \left( B \right)$$

$$VCO_{2\,LUNG} \, and\, VCO_{2\,ECMO} = Q \times \Delta_{v - a} CO_{2} ; \,VCO_{2\,total} = Q_{total} \times \Delta_{ao - v} CO_{2 } \quad \left( C \right)$$

1 $$\dot{Q}_{total} \times \Delta c_{v - ao} CO_{2} = \dot{Q}_{LUNG} \times \Delta c_{v - LA} CO_{2} + \dot{Q}_{ECMO} \times \Delta c_{v - pm} CO_{2} .$$

$\Delta c_{v - ao} CO_{2}$ is the difference between venous and aortal CO_2_ content, $\Delta c_{v - LA} CO_{2}$ is the difference between venous and left atrial CO_2_ content, $\Delta c_{v - pm} CO_{2}$ is the difference between venous and post membrane CO_2_ content (Fig. 1)^10^. By simultaneous measurement of exhaled CO_2_ at the lung and pCO_2_ at the ECMO gas outlet, respective fractions of carbon dioxide elimination ($\mathop {\text{V}}\limits^{.}$CO_2, ECMO_ and $\mathop {\text{V}}\limits^{.}$CO_2, LUNG_) can be calculated with simple means^10^. They should equal the blood carbon dioxide content difference in the respective segment. Rearrangement of formula 1 (formula D–F) proposes a proportional relationship between carbon dioxide elimination and respective blood flows:

$$Q_{total} \times \Delta_{ao - v} CO_{2} = Q_{LUNG} \times \Delta_{v - LA} CO_{2} + Q_{ECMO} \times \Delta_{v - pm} CO_{2} \quad \left( D \right)$$

$$\left( {Q_{LUNG} + Q_{ECMO} } \right) \times \Delta_{ao - v} CO_{2} = Q_{LUNG} \times \Delta_{v - LA} CO_{2} + Q_{ECMO} \times \Delta_{v - pm} CO_{2} \quad \left( E \right)$$

$$Q_{LUNG} \times \left( {\Delta_{ao - v} CO_{2} - \Delta_{v - LA} CO_{2} } \right) = Q_{ECMO} \times \left( {\Delta_{v - pm} CO_{2} - \Delta_{ao - v} CO_{2} } \right) \quad \left( F \right)$$

2 $$\dot{Q}_{LUNG} = \dot{Q}_{ECMO} \times \frac{{\left( {\Delta_{v - pm} CO_{2} - \Delta_{v - ao} CO_{2} } \right)}}{{\left( {\Delta_{v - ao} CO_{2} - \Delta_{v - LA} CO_{2} } \right)}} .$$

$\mathop {\text{V}}\limits^{.}$CO_2_ is the product of the differences in CO_2_ times the blood flow and formula 2 can therefore be simplified using the following assumptions: $\dot{V}CO_{2LUNG} \sim \Delta_{v - LA} CO_{2} ; \dot{V}CO_{2ECMO} \sim \Delta_{v - pm} CO_{2} ; \dot{V}CO_{2Total} \sim \Delta_{v - ao} CO_{2}$. The approximation signs are necessary, as $\mathop {\text{V}}\limits^{.}$CO_2_ is not only determined by the difference in veno-arterial CO_2_, but also by blood flow. As production and elimination are mathematical opposites, we use absolute values:

$$Q_{LUNG} = Q_{ECMO} \times \frac{{\left( {\left| {VCO_{2\,ECMO} } \right| - |VCO_{2\,\,total} |} \right)}}{{\left( {\left| {VCO_{2\,total} } \right| - |VCO_{2\,LUNG} |} \right)}}\quad \left( G \right)$$

3 $$\dot{Q}_{LUNG} = \dot{Q}_{ECMO} \times \frac{{\left| {\dot{V}CO_{2\,LUNG} } \right|}}{{|\dot{V}CO_{2\,ECMO} |}}.$$

The original Fick principle suggests that this deduction is also true for the oxygenation and O_2_ elimination ($\mathop {\text{V}}\limits^{.}$O_2_), such as:

4 $$\dot{Q}_{LUNG} = \dot{Q}_{ECMO} \times \frac{{\left| {\dot{V}O_{2\,LUNG} } \right|}}{{\left| {\dot{V}O_{2\,ECMO} } \right|}}.$$

The full deduction of these relationships has previously been described^10^.

Blood flow calculations were performed for measurements of $\mathop {\text{V}}\limits^{.}$O_2_ derived from blood content ($\mathop {\text{V}}\limits^{.}$O_2_), gaseous measurements of $\mathop {\text{V}}\limits^{.}$CO_2_, normalized at the lung for a $\mathop {\text{V}}\limits^{.}$/${\dot{\text{Q}}}$ of 1 ($\mathop {\text{V}}\limits^{.}$CO_2,GasNorm_) in order to assess the efficacy of the normalization procedure, gaseous measurements of $\mathop {\text{V}}\limits^{.}$ CO_2_, not normalized ($\mathop {\text{V}}\limits^{.}$CO_2,Gas_) in order to simulate clinical conditions and $\mathop {\text{V}}\limits^{.}$CO_2_ measurement derived from blood content ($\mathop {\text{V}}\limits^{.}$CO_2,Blood_).

### Normalization of $\mathop {\text{V}}\limits^{.}$CO_2_ towards a $\mathop {\text{V}}\limits^{.}$/${\dot{\text{Q}}}$ of 1

Blood flow determines the amount of CO_2_ transported towards the membrane lung, while ventilation determines the amount of CO_2_ eliminated over the respective membrane lung. When $\mathop {\text{V}}\limits^{.}$/${\dot{\text{Q}}}$ differs from 1, $\mathop {\text{V}}\limits^{.}$CO_2_ is predominantly determined by ventilation^11^ and thus does not correlate well with blood flow. This results in different venoarterial content differences across the ECMO and lung, which will introduce an error into Eqs. (3) and (4). As we aim to calculate blood flow through the lungs, we correct $\mathop {\text{V}}\limits^{.}$CO_2_ values on the ECMO towards a $\mathop {\text{V}}\limits^{.}$/${\dot{\text{Q}}}$ of 1^10^:

5 $$f\left( {\dot{V},\dot{Q}} \right) = \frac{{\dot{Q} \times \left( {\frac{{\dot{V}}}{{\dot{Q}}} + c} \right)}}{{\dot{V} \times \left( {1 + c} \right)}} = \frac{{\left( {\frac{{\dot{V}}}{{\dot{Q}}} + c} \right)}}{{\left( {1 + c} \right)}} \times \frac{1}{{\dot{V}/\dot{Q}}}.$$

The constant c was calculated from a venous blood gas sample [$c = \sigma_{{CO_{2} }} \times R \times T \times \left( {1 + K_{c} } \right)]$ as a function of temperature T, pH (K_c_), CO_2_ solubility ($\sigma_{{CO_{2} }} )$ and the gas constant R^20^. The normalization essentially allows to calculate the $\mathop {\text{V}}\limits^{.}$CO_2_ values only dependent of blood flow and independent of ventilation, i.e. for any given $\mathop {\text{V}}\limits^{.}$/${\dot{\text{Q}}}$-ratio, the normalization calculates the $\mathop {\text{V}}\limits^{.}$CO_2_ for a $\mathop {\text{V}}\limits^{.}$/${\dot{\text{Q}}}$ of 1. In a clinical setting the $\mathop {\text{V}}\limits^{.}$/${\dot{\text{Q}}}$ ratio at the ECMO is known, therefore $\mathop {\text{V}}\limits^{.}$CO_2_ at the ECMO is always normalized.

### Calculations of shunt and its impact on blood flow calculations

Pulmonary right-to-left shunt fraction was calculated as shunt blood flow divided by total lung flow. The relative error in blood flow calculations resulting from varying shunt was calculated as true blood flow minus calculated blood flow divided by true blood flow. Using the Berggren shunt equation with O_2_ contents and CO_2_ contents, we calculated the estimated shunt fractions.

6 $$\% Shunt = \dot{Q}s/\dot{Q}t = \frac{{\Delta c_{pL - LA} O_{2} /CO_{2} }}{{\Delta c_{pL - v} O_{2} /CO_{2} }}.$$

The error produced by the shunt fraction is calculated as:

7 $$Error = \frac{{\dot{Q}_{measured} - \dot{Q}_{calculated} }}{{\dot{Q}_{measured} }} .$$

### Calculations of CO_2_ and O_2_ content and of $\mathop {\text{V}}\limits^{.}$CO_2_ and $\mathop {\text{V}}\limits^{.}$O_2_

Blood CO_2_ content (cCO_2_) was calculated for each sampling port with the method of Dash^21,22^. O_2_ content (CO_2_) was calculated for each sampling port using formula (7) (pO_2_: O_2_ partial pressure [mmHg], sO_2_: Saturation, Hb: Hemoglobin [g/L]):

8 $$cO_{2} = 1.36 \times Hb \times \frac{{sO_{2} }}{100} + 0.003 \times pO_{2} .$$

$\mathop {\text{V}}\limits^{.}$O_2_ was calculated by multiplying the arterio-venous-$\mathop {\text{V}}\limits^{.}$enous O_2_ blood content difference with blood flow. $\mathop {\text{V}}\limits^{.}$CO_2_ was calculated either from venous-arterial CO_2_ content difference multiplied by blood flow ($\mathop {\text{V}}\limits^{.}$CO_2, Blood_) or from gaseous measurements by multiplying the exhausted CO_2_ fraction times the sweep gas flow ($\mathop {\text{V}}\limits^{.}$CO_2, Gas_). The relationship between $\mathop {\text{V}}\limits^{.}$CO_2, Gas_ and $\mathop {\text{V}}\limits^{.}$CO_2, Blood_ was assessed using multiple linear regression with blood flow (${\dot{\text{Q}}}$) and differences in CO_2_ content (∆cCO_2_) as independent variables and $\mathop {\text{V}}\limits^{.}$CO_2, Gas_ as dependent variables such as:

9 $$\dot{V}CO_{2\,Gas} = Intercept + \beta_{1} \times \Delta cCO_{2} + \beta_{2} \times Q + \beta_{3} \times \Delta cCO_{2} \times Q .$$

### Statistical analysis

For statistical, mathematical and graphical analysis, we used Matlab R2020a (MathWorks, Natick, Massachusetts, USA with an extension for Bland Altman Plots under creative commons license^23^). Data were not normally distributed and thus presented as median with interquantile [0.25–0.75] ranges. Correlation between methods was assessed with linear regression and method agreement with Bland–Altman analysis^24,25^. p < 0.05 was considered significant with two-tailed testing. Linear regression was performed using the least square fit method. Correlation coefficients were calculated using Pearson’s square (r^2^). The least significant change of a method was calculated according to standard methods^26^. Multiple linear regression was used to assess the relationship between $\mathop {\text{V}}\limits^{.}$CO_2_, blood flow and differences in CO_2_ content.

## Results

### Baseline

At the beginning of each experimental step, mixed venous saturation was 87.6 [82.7–88.5] % and hemoglobin was 9 [8.5–9.1] g/dl, corresponding to a venous O_2_ content of 10.9 [9.7–11.0] ml/100 ml of blood and a mixed venous pCO_2_ of 48.6 [48.1–51.7] mmHg corresponding to a venous CO_2_ content of 68.4 [66.9–89.4] ml/100 ml of blood. Arterial oxygen saturation was 99.5 [98.7–100.0] % corresponding to an arterial O_2_ content of 12.8 [11.7–13.1] ml/100 ml of blood. Arterial pCO_2_ was 33.8 [32.3–35.9] mmHg corresponding to an arterial CO_2_ content of 59.9 [58.7–78.3] ml/100 ml of blood. Arterial and venous pH were 7.39 [7.34–7.47] and 7.28 [7.24–7.36], respectively.

#### ECMO (Oxy_ECMO_)

${\dot{\text{Q}}}$_ECMO_ was 1947 [1786–1942] ml/min. $\mathop {\text{V}}\limits^{.}$CO_2, Gas, ECMO_ was 82.4 [78.4–95.4] ml/min and $\mathop {\text{V}}\limits^{.}$CO_2, Blood, ECMO_ was 128.0 [114.7–185.3] ml/min. $\mathop {\text{V}}\limits^{.}$O_2, Blood, ECMO_ was 39.5 [32.4–43.3] ml/min. $\mathop {\text{V}}\limits^{.}$_ECMO_ was kept steady between 1.4 and 1.5 l/min for the maneuvers with a varying $\mathop {\text{V}}\limits^{.}$/${\dot{\text{Q}}}$_ECMO_ (steps 1b) and followed blood flow in the remaining maneuvers. The correction factor c used in the normalization of $\mathop {\text{V}}\limits^{.}$/${\dot{\text{Q}}}$ was 10.4 [9.5–12.5].

#### Simulated lung (Oxy_Lung_)

${\dot{\text{Q}}}$_Lung_ was 340 [296 to 464] ml/min with a $\mathop {\text{V}}\limits^{.}$CO_2, Gas, Lung_ of 45.5 [43.1–48.7] ml/min while $\mathop {\text{V}}\limits^{.}$CO_2, Blood, Lung_ was 82.0 [69.6–88.8] ml/min. $\mathop {\text{V}}\limits^{.}$O_2, Blood, Lung_ was 7.4 [5.0–9.3] ml/min. Ventilation at the lung ($\mathop {\text{V}}\limits^{.}$_Lung_) was set between 1.4 and 1.5 l/min and remained unchanged.

#### Metabolic chamber (Oxy$_{{\mathop {\text{V}}\limits^{.} {\text{CO}}_{2} /\mathop {\text{V}}\limits^{.} {\text{O}}_{2} }}$)

Total flow was 2254 [1947–2409] ml/min. N_2_ gas flow was kept between 4 and 6 l/min while CO_2_ gas flow was between 300 and 500 ml/min, corresponding to a $\mathop {\text{V}}\limits^{.}$CO_2, Blood, Metabolic Chamber_ of 203.5 [173.4–230] ml/min and a $\mathop {\text{V}}\limits^{.}$O_2, Blood, Metabolic Chamber_ of 44.4 [39.6–50.2] ml/min.

### Maneuvers and blood flow reductions during step one and two

#### ECMO (Oxy_ECMO_)

In each maneuver, ECMO blood flow was reduced to 25.2% [23.0–25.9] % of baseline. $\mathop {\text{V}}\limits^{.}$O_2, Blood, ECMO_ correlated highly with these reductions in ECMO blood flow ($\mathop {\text{V}}\limits^{.}$O_2, Blood, ECMO_ = 0.02 × ECMO Blood Flow + 5.4, r^2^ = 0.785, p < 0.001). $\mathop {\text{V}}\limits^{.}$CO_2, GasNorm, ECMO_ showed a high correlation with ECMO blood flow ($\mathop {\text{V}}\limits^{.}$CO_2, GasNorm, ECMO_ = 0.05 × ECMO Blood Flow + 1.3, r^2^ = 0.963, p < 0.001). Correlation decreased with $\mathop {\text{V}}\limits^{.}$CO_2, Gas, ECMO_ ($\mathop {\text{V}}\limits^{.}$CO_2, Gas, ECMO_ = 0.04 × ECMO Blood Flow + 16.2, r^2^ = 0.796, p < 0.001). $\mathop {\text{V}}\limits^{.}$CO_2, Blood, ECMO_ and ECMO Blood flow showed only weak correlations ($\mathop {\text{V}}\limits^{.}$CO_2, Blood, ECMO_ = 0.07 × ECMO Blood Flow + 25.8, r^2^ = 0.333, p < 0.001).

#### Simulated lung (Oxy_Lung_)

Lung blood flow increased to 1417 [1234–1540] ml/min in Step 1a and 1b, with a shunt of 20.5 [18.6–22.2] % in the 20% shunt maneuvers and a shunt of 35.3 [32.7–39.7] % in the 40% shunt maneuvers. In step 2a and 2b, lung blood flow increased to a median of 831 [660 to 1254] ml/min.

$\mathop {\text{V}}\limits^{.}$O_2, Blood, Lung_ showed high correlations with these increases in lung blood flow ($\mathop {\text{V}}\limits^{.}$O_2, Blood, Lung_ = 0.02 × Lung Blood Flow + 3.7, r^2^ = 0.761, p < 0.001). $\mathop {\text{V}}\limits^{.}$CO_2, GasNorm, Lung_ showed a high correlation with lung blood flow ($\mathop {\text{V}}\limits^{.}$CO_2, GasNorm, Lung_ = 0.05 × Lung Blood Flow − 4.2, r^2^ = 0.986, p < 0.001). With $\mathop {\text{V}}\limits^{.}$CO_2, Gas, Lung_ correlation decreased ($\mathop {\text{V}}\limits^{.}$CO_2, Gas, Lung_ = 0.02 × Lung Blood Flow + 40.3, r^2^ = 0.673, p < 0.001). Correlations between lung blood flow and $\mathop {\text{V}}\limits^{.}$CO_2, Blood, Lung_ decreased further ($\mathop {\text{V}}\limits^{.}$CO_2, Blood, Lung_ = 0.07 × Lung Blood Flow + 60.3, r^2^ = 0.189, p = 0.006).

#### Metabolic chamber (Oxy$_{{\mathop {\text{V}}\limits^{.} {\text{CO}}_{2} /\mathop {\text{V}}\limits^{.} {\text{O}}_{2} }}$)

During Step 1a and Step 1b, blood flow in the metabolic chamber was 2359 [2004–2487] ml/min and during step 2a and 2b, flow in the metabolic chamber was 1770 [1406–2068] ml/min. Calculating total CO_2_ production/elimination as $\mathop {\text{V}}\limits^{.}$CO_2, Blood, Metabolic Chamber_ minus the sum of $\mathop {\text{V}}\limits^{.}$ CO_2, Blood, Lung_ and $\mathop {\text{V}}\limits^{.}$CO_2, Blood, ECMO_ showed a median value of 6.1 [− 2.0 to 13.1] ml/min. Calculating total O_2_ consumption and transfer as the sum of $\mathop {\text{V}}\limits^{.}$O_2, Blood, Lung_ and $\mathop {\text{V}}\limits^{.}$O_2, Blood, ECMO_ minus $\mathop {\text{V}}\limits^{.}$O_2_, _Blood, Metabolic Chamber_ showed a median value of 2.6 [0.3–4.9] ml/min.

**Figure 3:**
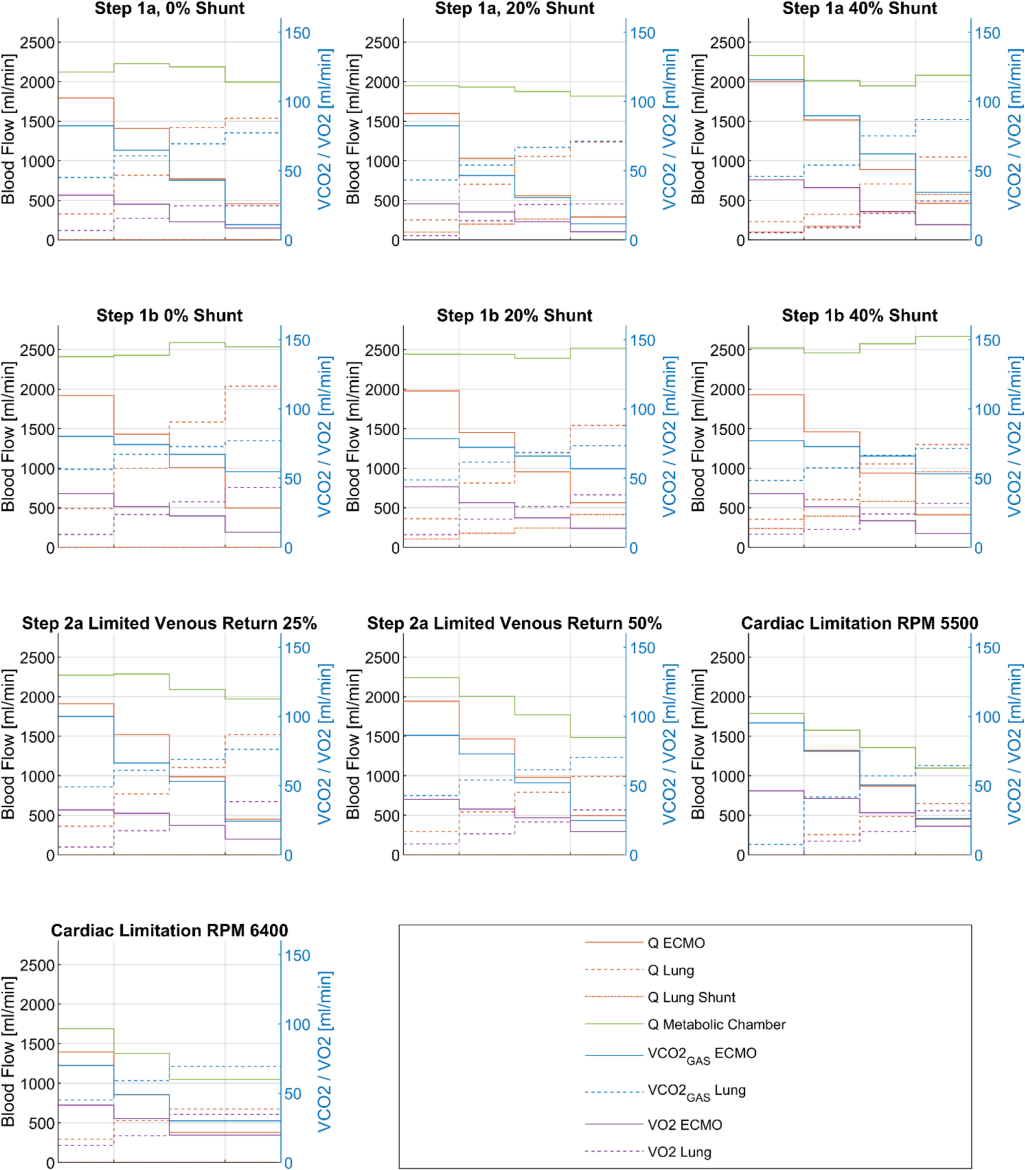
Stairs plot showing measured data from performed experiment with manoeuvres Step 1a, Step 1b, Step 2a and Step 2b. Note that in Step 2b, Cardiac Limitation there only 3 data points due to pump failure.

All measured blood flows as well as $\mathop {\text{V}}\limits^{.}$CO_2Gas_ and $\mathop {\text{V}}\limits^{.}$O_2_ values are presented in Fig. 3. Based on these measurements, we calculated simulated pulmonary blood flow using $\mathop {\text{V}}\limits^{.}$O_2_, $\mathop {\text{V}}\limits^{.}$CO_2, Gas Norm_, $\mathop {\text{V}}\limits^{.}$CO_2, Gas_ and $\mathop {\text{V}}\limits^{.}$CO_2, Blood_.

### Blood flow calculations

#### Based on $\mathop {\text{V}}\limits^{.}$O_2_

**Figure 4:**
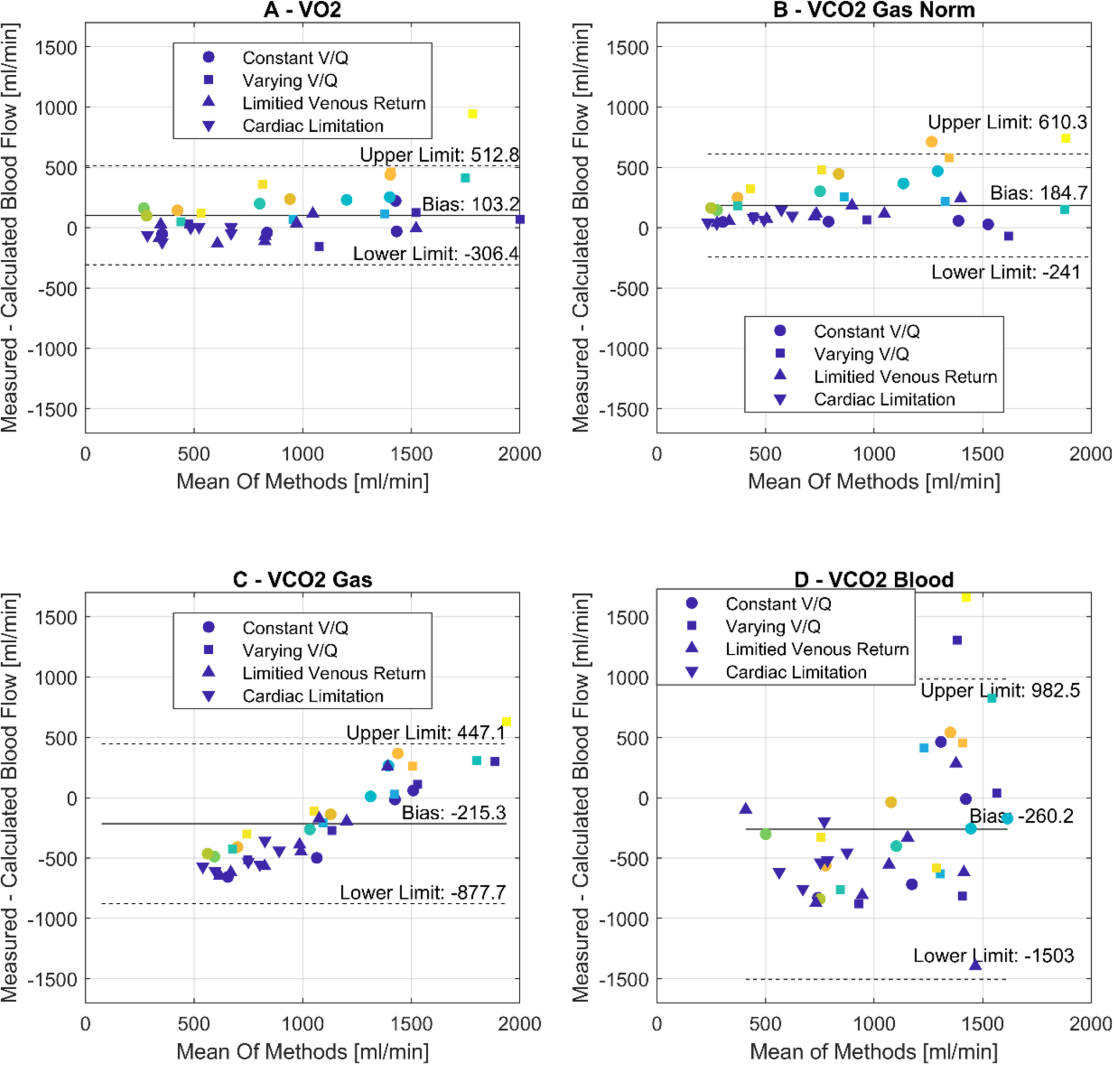
Blood flow calculations using $\mathop {\text{V}}\limits^{.}$O_2_ (**A**), $\mathop {\text{V}}\limits^{.}$CO_2GasNorm_ normalized for ECMO and Lung to a $\mathop {\text{V}}\limits^{.}$/${\dot{\text{Q}}}$ of 1 (**B**), $\mathop {\text{V}}\limits^{.}$CO_2Gas_ not normalized at the Lung (**C**) and $\mathop {\text{V}}\limits^{.}$CO_2Blood_ (**D**). Mean and upper/lower are depicted within each graph. Circles, squares, triangles up and down refer to the 4 different maneuvers performed. Color indicates shunt with blue representing 0% shunt and yellow representing maximum shunt (42.4%).

Linear regression showed high correlations between measured and calculated blood flow through the lung circuit using $\mathop {\text{V}}\limits^{.}$O_2, Blood_ values (Calculated Blood Flow = 0.76 × measured Blood Flow + 120 ml/min, r^2^ = 0.88, p < 0.001, Fig. 4A). The positive bias (103 ml/min) indicated an underestimation of measured blood flow. The least significant change was 95 ml/min. Exclusion of shunt values (> 1%) increased the accuracy of the method and cancels underestimation (Calculated Blood flow = 1.08 × measured Blood Flow − 80.615 ml/min, r^2^ 0.97, p < 0.001, Bias − 12 ml/min [Upper Limit 167 ml, Lower Limit − 191 ml/min]).

#### Based on $\mathop {\text{V}}\limits^{.}$CO_2, GasNorm_

Linear regression showed high correlations for simulated ${\dot{\text{Q}}}$_Lung_ calculations using $\mathop {\text{V}}\limits^{.}$CO_2GasNorm_, which is corrected for a $\mathop {\text{V}}\limits^{.}$/${\dot{\text{Q}}}$ of 1 at Oxy_Lung_ as well as Oxy_ECMO_ (Calculated Blood Flow = 0.88 × measured Blood Flow − 72 ml, r^2^ = 0.85, p < 0.001, Fig. 4B). The calculated bias (185 ml/min) is positive, indicating an underestimation of measured blood flow. The least significant change was 100 ml/min. Exclusion of shunt values (> 1%) increased the accuracy of the method (Calculated Blood flow = 0.87 × measured Blood Flow − 157.76 ml/min, r^2^ 0.96, p < 0.001, Bias 56 ml/min [Upper Limit 299 ml, Lower Limit − 187 ml/min]).

#### Based on $\mathop {\text{V}}\limits^{.}$CO_2, Gas_

Regression coefficients decreased, when $\mathop {\text{V}}\limits^{.}$CO_2, Gas_ was only normalized at Oxy_ECMO_ but not at Oxy_Lung_ to calculate simulated blood flows through the lung circuit, as would be the case in a clinical setting (Calculated Blood Flow = 0.45 × measured Blood Flow + 728 ml/min, r^2^ = 0.79, p < 0.001, Fig. 4C). The bias (− 268 ml/min) is negative and the lack of normalization leads to a significant relationship between the mean and the difference of methods (Difference of Method = 0.69 × Bias − 932, r^2^ = 0.72, p < 0.001), leading to an overestimation of blood flow at low flows and an underestimation of blood flows at high flows. This corresponds to the change in $\mathop {\text{V}}\limits^{.}$/${\dot{\text{Q}}}$, e.g. low $\mathop {\text{V}}\limits^{.}$/${\dot{\text{Q}}}$ at high blood flow and high $\mathop {\text{V}}\limits^{.}$/${\dot{\text{Q}}}$ at low blood flow. Exclusion of shunt values (> 1%) increased the slope of the regression but does otherwise not affect the accuracy of the method (Calculated Blood flow = 2.14 × measured Blood Flow + 1671 ml/min, r^2^ 0.90, p < 0.001, Bias − 333 ml/min [Upper Limit 256 ml/min, Lower Limit − 921 ml/min]).

#### Based on $\mathop {\text{V}}\limits^{.}$CO_2, Blood_

Blood flow calculations with $\mathop {\text{V}}\limits^{.}$CO_2, Blood_ show high inaccuracy with no significant relationship between measured lung circuit flow and calculated lung circuit flow (Calculated Blood Flow = 0.08 × measured Blood Flow + 1150 ml, r^2^ = 0.01, p = 0.49, Fig. 4D), corresponding to a bias at − 268 ml/min with wide limits of agreement (− 1503 ml to 967 ml/min). There is no improvement in accuracy through exclusion of shunt values (Calculated Blood flow = 0.24 × measured Blood Flow + 538 ml, r^2^ 0.04, p = 0.4, Bias − 409 ml/min [Upper Limit 724 ml/min, Lower Limit − 1541 ml/min]).

### Impact of shunt

#### $\mathop {\text{V}}\limits^{.}$O_2, Blood, Lung_

**Figure 5:**
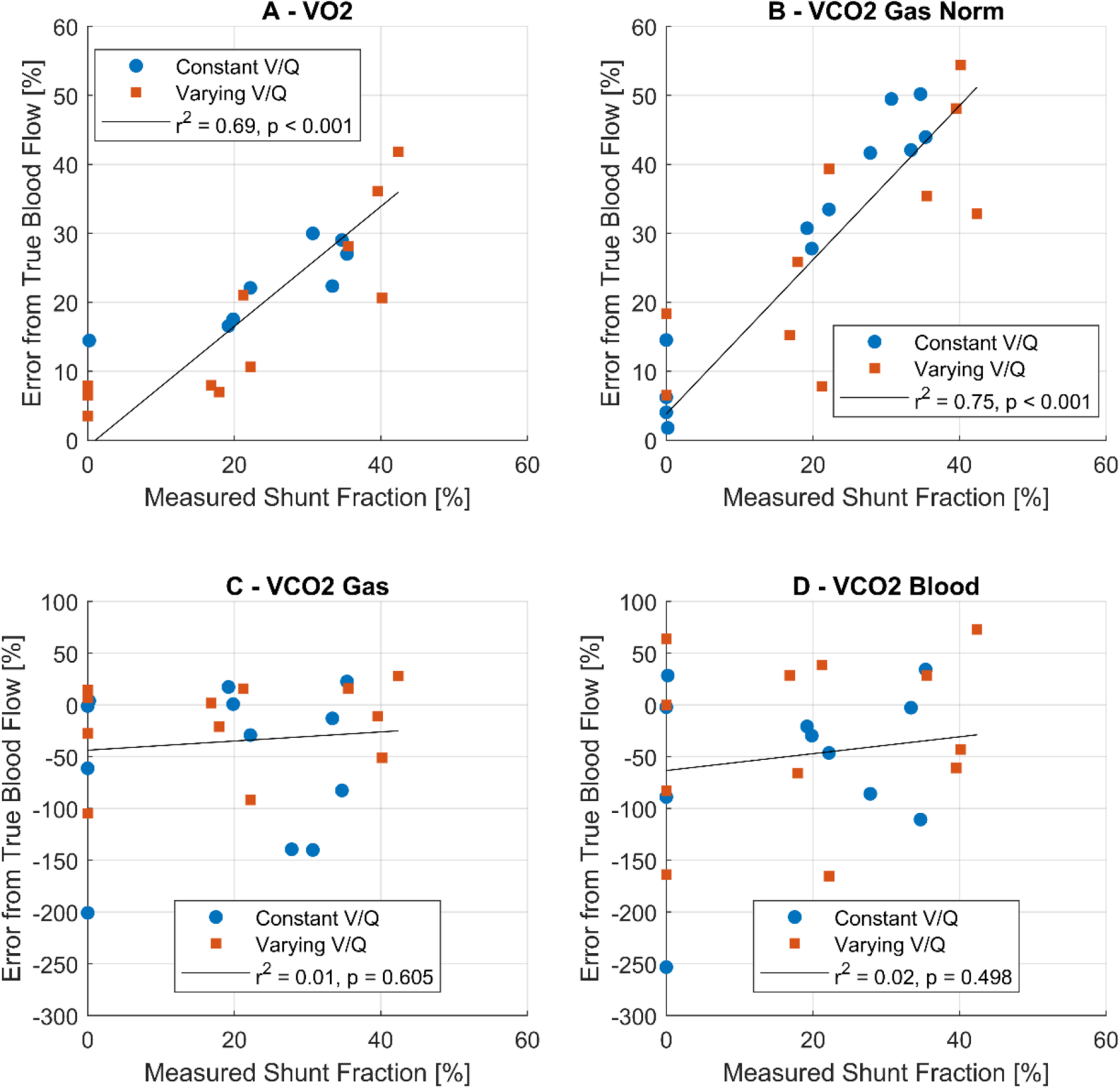
Influence of shunt on blood flow calculations using $\mathop {\text{V}}\limits^{.}$O_2_ (**A**), $\mathop {\text{V}}\limits^{.}$ CO_2GasNorm_ normalized for ECMO and Lung to a $\mathop {\text{V}}\limits^{.}$/${\dot{\text{Q}}}$ of 1 (**B**), $\mathop {\text{V}}\limits^{.}$CO_2Gas_ not normalized at the Lung (**C**) and $\mathop {\text{V}}\limits^{.}$CO_2Blood_ (**D**). Maneuvers simulating cardiac limitation and limited venous return are excluded, because they did not include any shunt per protocol.

There is a significant relationship between shunt values and the produced error from measured blood flow (Error = 0.87 × Shunt − 0.9%, r^2^ = 0.7, p < 0.001, Fig. 5A).

#### $\mathop {\text{V}}\limits^{.}$CO_2, GasNorm, Lung_

The influence of shunt on blood flow calculations is not only present in calculations using $\mathop {\text{V}}\limits^{.}$O_2_, _Blood, Lung_ values but also $\mathop {\text{V}}\limits^{.}$CO_2, GasNorm, Lung_ values (Error = 1.11 × Shunt − 3.8%, r^2^ = 0.75, p < 0.001, Fig. 5B).

Using classical Berggren shunt equations, we can show that determining shunt fractions is possible with both CO_2_ and O_2_ contents, with more variance in CO_2_ calculations (**O**_**2**_: Calculated shunt = 0.88 × Set Shunt + 0.33%, r^2^ = 0.792, p < 0.001; CO_2_: Calculated shunt = 0.96 × Set Shunt + 0.35%, r^2^ = 0.653, p < 0.001). Comparing set shunt to calculated shunt shows a bias of 1.08% for O_2_ derived shunt (Lower Limit: − 13.03%, Upper Limit 15.2%) and a bias of 0.08% for CO_2_ derived shunt (Lower Limit: − 21.15%, Upper Limit 21.31%).

Blood flow calculations using $\mathop {\text{V}}\limits^{.}$CO_2, Gas_ (Error = 0.44 × Shunt − 43.7%, r^2^ = 0.01, p = 0.605, Fig. 5C) and $\mathop {\text{V}}\limits^{.}$CO_2, Blood_ (Error = 0.81 × Shunt − 63.3%, r^2^ = 0.02, p = 0.498, Fig. 5D) do not show enough accuracy in order to determine the influence of shunt.

### Relationship between CO_2_ content in blood, blood flow and $\mathop {\text{V}}\limits^{.}$CO_2_, Gas, calculated for Oxy_ECMO_ and Oxy_Lung_

**Figure 6:**
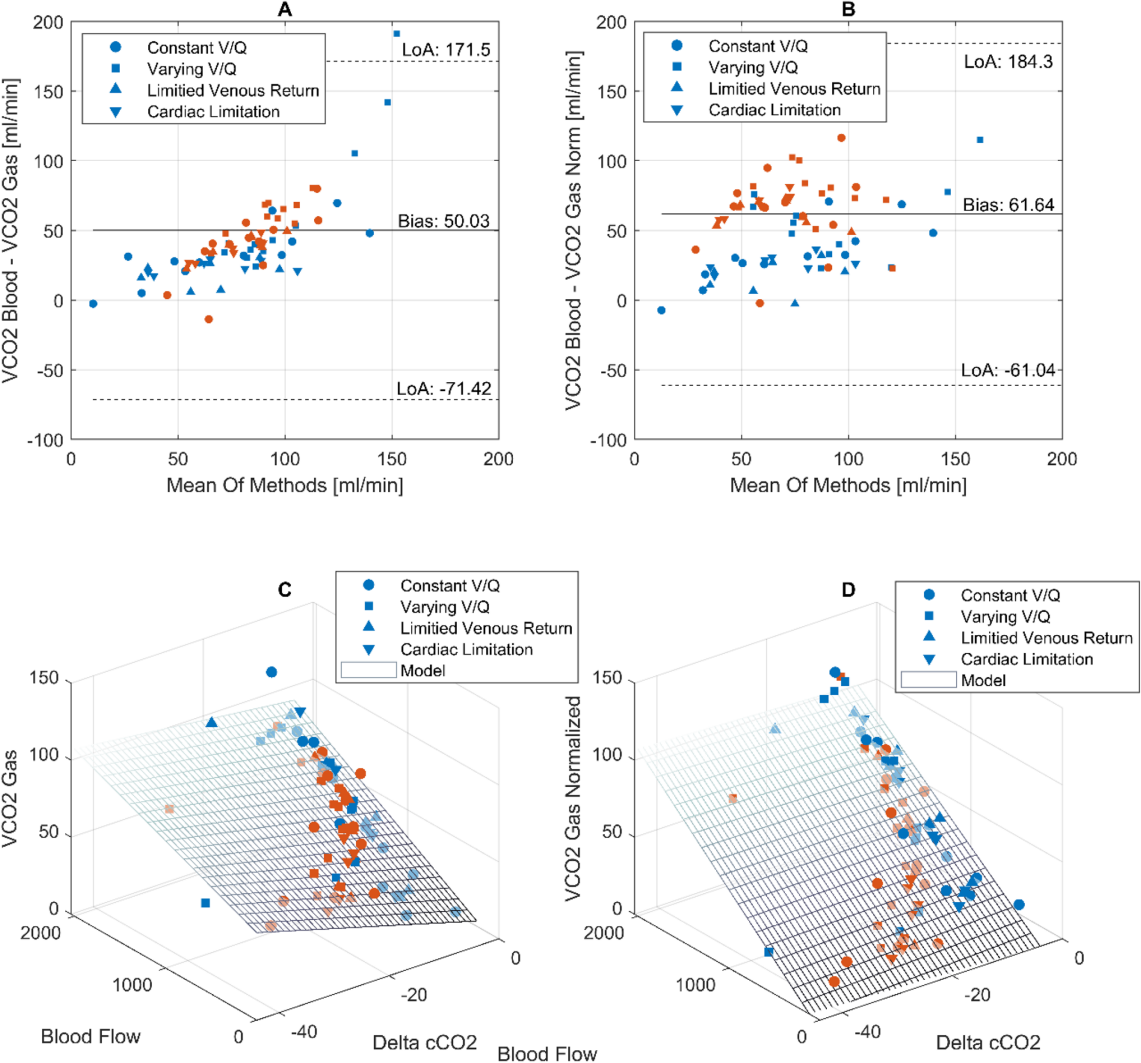
(**A**) Bland–Altman plot for $\mathop {\text{V}}\limits^{.}$CO_2Blood_ against $\mathop {\text{V}}\limits^{.}$CO_2Gas_. (**B**) Bland–Altman plot for $\mathop {\text{V}}\limits^{.}$CO_2Blood_ against $\mathop {\text{V}}\limits^{.}$CO_2GasNorm_. Multiple regression analysis showing the relationship between Blood Flow, Delta in CO_2_ Content and $\mathop {\text{V}}\limits^{.}$CO_2Gas_ (**C**) and $\mathop {\text{V}}\limits^{.}$CO_2GasNorm_ (**D**). Regression coefficients can be found in Table 1. Values from Oxy_ECMO_ are blue while values from Oxy_Lung_ are in orange color.

$\mathop {\text{V}}\limits^{.}$CO_2, Blood_ overestimates $\mathop {\text{V}}\limits^{.}$CO_2, Gas_ with a bias of 50 ml/100 ml blood (Fig. 6A). The wide limits of agreement (Lower Limit − 71 ml/100 ml blood, Upper Limit 171 ml/100 ml blood) show that $\mathop {\text{V}}\limits^{.}$CO_2, Blood_ is inconsistent and unreliable. $\mathop {\text{V}}\limits^{.}$CO_2GasNorm_ shows similar bias (62 ml/100 ml blood) and limits of agreement (− 61 to 184 ml/100 ml blood), but Fig. 6B shows that the spread of data is more uniform. Exclusion of shunt values (> 1%) reduced bias to 24 ml/100 ml blood [− 32 to 80 ml/100 ml blood] for $\mathop {\text{V}}\limits^{.}$CO_2Gas_ and a bias of 32 ml/100 ml blood [− 28 to 91 ml/100 ml blood].

$\mathop {\text{V}}\limits^{.}$CO_2, Blood_ is the product of the difference in CO_2_ content (Delta cCO_2_) and blood flow. Multiple linear regression shows a high significance between $\mathop {\text{V}}\limits^{.}$CO_2, Gas_, $\mathop {\text{V}}\limits^{.}$CO_2, GasNorm_ and these components of $\mathop {\text{V}}\limits^{.}$CO_2, Blood_ with both high r^2^ and significance (Table 1, Fig. 6C,D). The multiple linear regression on $\mathop {\text{V}}\limits^{.}$CO_2GasNorm_ results in Delta cCO_2_ being non-significant.

**Table 1:** Multiple linear regression according to model specified in formula 8 for $\mathop {\text{V}}\limits^{.}$CO_2Gas_ (r^2^ = 0.743, p < 0.001) and $\mathop {\text{V}}\limits^{.}$CO_2GasNorm_ (r^2^ = 0.974, p < 0.001).

	$\mathop {\text{V}}\limits^{.}$CO_2Gas_—Coefficient	$\mathop {\text{V}}\limits^{.}$CO_2Gas_—p value	$\mathop {\text{V}}\limits^{.}$CO_2GasNorm_—coefficient	$\mathop {\text{V}}\limits^{.}$CO_2GasNorm_—p value
Intercept	12.397	0.019	0.75	0.763
β_1_—Delta cCO_2_ (ml/100 min)	− 1.007	< 0.001	0.17	0.170
β_2_—Blood Flow (ml)	0.039	< 0.001	0.05	< 0.001
β_3_—Delta cCO_2_ × blood flow	0.0003	0.309	− 0.0002	0.242

## Discussion

Our simulation of two competing blood circuits (ECMO and lung) with a deoxygenation and carboxylation unit (metabolic chamber) was able to reach physiologically representative parameters regarding gas exchange and blood content of carbon dioxide and oxygen. The blood flows in this experiment are adequate for the pediatric oxygenators and the simulated $\mathop {\text{V}}\limits^{.}$O_2_. As the main result, we could calculate simulated pulmonary blood flow with high accuracy and precision and correlations using $\mathop {\text{V}}\limits^{.}$CO_2, GasNorm_ and $\mathop {\text{V}}\limits^{.}$O_2, Blood_ values. This confirms our main hypothesis that gas exchange may be used for blood flow calculations in extracorporeal circuits. The underlying physiological principles and mass conservation show that CO_2_ production and O_2_ consumption must be in equilibrium in two competing systems with two circuits and oxygenators, which allows calculation of blood flow within certain limits^10^. The mass balance equations do not necessarily pose a need for the calculation with absolute values as suggested by Eqs. (3) and (4). The absolute values make the interpretation of results easier, since they cancel out directional (I. E. elimination or production) effects of gas exchange on the direction of calculated blood flow, as we have previously published^10^. The accuracy of these flow calculations is impaired by high shunt and $\mathop {\text{V}}\limits^{.}$/${\dot{\text{Q}}}$ mismatch. Shunted blood will not participate in gas exchange and is therefore not detected by our method. We showed that the simulated pulmonary shunt has a linear relationship to the difference in CO_2_ content as it does with O_2_ content. This shunt contributes to inaccuracy in our model. A shunt of 100%, which is possible upon initiation of ECMO therapy, would therefore produce a calculated blood flow of 0 ml/min. The linear regressions of Fig. 5A,B estimate an error of 86.7% and 107.2% respectively for an assumed shunt of 100%, which confirms this assumption. In the three-compartment model of the lung^27^, shunt or venous admixture is seen as the cause of hypoxemia, while excessive dead space ventilation with exhaustion of respiratory reserves explains hypercapnia^27^. Our results indicate that increased arterial pCO_2_ could be caused by shunt as well, when the alveolar minute ventilation stays constant, as it would be the case during controlled mechanical ventilation.

Blood flow through the oxygenator and $\mathop {\text{V}}\limits^{.}$CO_2, Gas_ and $\mathop {\text{V}}\limits^{.}$O_2, Blood_ show a strong correlation. Mass conservation implies that Eqs. (3) and (4) are only true if two prerequisites are met: First, the inflow content into both circuits needs to be equal and second, the difference in gas content across the lung and the ECMO must be the same. If $\mathop {\text{V}}\limits^{.}$/${\dot{\text{Q}}}$ ratio at the ECMO and the lung are not equal, the second prerequisite is not met due to the influence of ventilation on the veno-arterial content difference^11,12^. Therefore, the best result using carbon dioxide based calculations is achieved with $\mathop {\text{V}}\limits^{.}$CO_2, GasNorm_ for both lung and ECMO, where content differences are normalized for inequalities introduced by ventilation^10^. High $\mathop {\text{V}}\limits^{.}$/Q will lead to an overestimation of pulmonary blood flow and low $\mathop {\text{V}}\limits^{.}$/${\dot{\text{Q}}}$ will lead to an underestimation (Fig. 5C).

The differences in the blood contents of CO_2_ or O_2_ share a relationship with $\mathop {\text{V}}\limits^{.}$CO_2_ and $\mathop {\text{V}}\limits^{.}$O_2_ in the gas phase, which represents the physiological background to replace the blood content used in the original Fick description with $\mathop {\text{V}}\limits^{.}$CO_2_ and $\mathop {\text{V}}\limits^{.}$O_2_ in the gas phase (formula 3 and 4). The slope of the grids in Fig. 6C represent the proportionality between the differences in blood gas content and $\mathop {\text{V}}\limits^{.}$CO_2, Gas_. Both models for the simple as well as the normalized gas measurement show high goodness of fit. The model for $\mathop {\text{V}}\limits^{.}$CO_2Gas_ shows the delta in CO_2_ content as well as blood flow as significant determinants whereas after the normalization, only blood flow remains as a significant factor. If blood flow remains steady, different $\mathop {\text{V}}\limits^{.}$CO_2_ values will result from different ventilation settings, i.e. varying $\mathop {\text{V}}\limits^{.}$/${\dot{\text{Q}}}$ ratios are traversed. This results in varying blood CO_2_ content differences for the same blood flow. As a side note, this may have a major effect on v-a PCO_2_ gradients, which are proposed for monitoring of the microcirculation^28^.

The normalization shown in Fig. 6D discards the effect of the difference in CO_2_ content and fully reestablishes the relationship in Eqs. (3) and (4). This improves the accuracy of our blood flow estimations, since varying $\mathop {\text{V}}\limits^{.}$/${\dot{\text{Q}}}$ ratios are corrected for and the normalization reestablishes equal differences in gas content across the ECMO and the lung. This method for normalization was previously described^10^.

Such a normalization seems unnecessary for $\mathop {\text{V}}\limits^{.}$O_2_, as oxygen is primarily transported by binding to hemoglobin (formula 7). As long as post oxygenator saturation reaches 100%, $\mathop {\text{V}}\limits^{.}$O_2_ is independent of $\mathop {\text{V}}\limits^{.}$/${\dot{\text{Q}}}$ ratio and represents blood flow, thus the difference and gas content across the ECMO and the lung are the same. Only if hemoglobin were to become incompletely saturated, ventilation would have an influence on $\mathop {\text{V}}\limits^{.}$O_2_. An increase in F_i_O_2_ will correct this phenomenon, unless a functional shunt exists^29^.

Blood flow calculations for $\mathop {\text{V}}\limits^{.}$CO_2, Blood_ are not reliable because there is no normalization applied and calculating the CO_2_ blood content can be challenging, since mathematical models do not represent the underlying physical chemistry completely^30,31^. Gas measurements are easily done and calculating $\mathop {\text{V}}\limits^{.}$CO_2_ from exhaust capnography is simple, readily available and reliable^32^. The multiple linear regression shows a strong relationship between $\mathop {\text{V}}\limits^{.}$CO_2Gas_ and $\mathop {\text{V}}\limits^{.}$CO_2Blood_, which proves the underlying physiological principle.

In our previously published animal series with this new method^10^, we used the differences in $\mathop {\text{V}}\limits^{.}$CO_2_ and blood flow during weaning, as the experimental setup did not allow to reach a steady state. In the present simulation study—using a highly controllable environment—such a steady state could be reached quickly. Therefore, we validated our method using these steady state conditions rather than differences in gas change during weaning. We interpret the small content differences in the $\mathop {\text{V}}\limits^{.}$CO_2, Blood_ and $\mathop {\text{V}}\limits^{.}$O_2, Blood_ at the metabolic chamber and the lung and membrane contents as limits of the calculation models rather than expressions of non-steady state.

Transferring our findings to a clinical setting would imply that the estimations of pulmonary blood flow during ECMO weaning are possible using F_i_O_2_, exhaust pCO_2_/pO_2_, $\mathop {\text{V}}\limits^{.}$enous pH, ECMO blood flow, ECMO ventilation, lung alveolar ventilation and lung dead space. The two latter parameters are readily available using volumetric capnography^33,34^, and all of these parameters can easily be measured in an intensive care unit where ECMO therapy is performed. Our method is limited by high shunt and $\mathop {\text{V}}\limits^{.}$/${\dot{\text{Q}}}$ mismatch, which we can only partially correct for. In a clinical setting, the $\mathop {\text{V}}\limits^{.}$/${\dot{\text{Q}}}$ ratio at the lung would remain unknown, but $\mathop {\text{V}}\limits^{.}$CO_2Gas_ at the lung might be corrected using alternative estimations of $\mathop {\text{V}}\limits^{.}$/${\dot{\text{Q}}}$. Multiple approaches exist such as MIGET^35^, electrical impedance tomography and positron emission tomography^36^, even though they are not in widespread clinical use. High precision measurements of gas exchange at the lung might increase the precision of our approach^37^. We would therefore suggest that our method will have the most benefit during ECMO weaning, when in general the patient’s $\mathop {\text{V}}\limits^{.}$/${\dot{\text{Q}}}$ mismatch has improved.

This study has multiple limitations: Firstly, we calculate simulated pulmonary blood flow using a high fidelity in-vitro simulation and the transfer to a clinical setting might be limited. Secondly, we have not performed gaseous measurements of $\mathop {\text{V}}\limits^{.}$O_2_. However, from a physiological point of view these should correlate well with measurements of $\mathop {\text{V}}\limits^{.}$O_2_ in the blood phase. Thirdly, our calculations of VCO_2, Blood_ show a high bias and wide limits of agreement with $\mathop {\text{V}}\limits^{.}$CO_2, Gas_. This is in contrast to recent findings^38^, where $\mathop {\text{V}}\limits^{.}$CO_2, Blood_ correlated well with $\mathop {\text{V}}\limits^{.}$CO_2, Gas_. In our model, this mismatch may be owed to the fact that we used a red cell suspension and not whole blood. The missing serum fraction may have influenced the content calculation^31^. Figure 6 suggests that the differences could also be caused by $\mathop {\text{V}}\limits^{.}$/${\dot{\text{Q}}}$ mismatch and shunt, because calculations excluding shunt shows its influence on these differences. Fourthly, the here proposed method assesses the heart and lung function of a patient simultaneously as a functional unit. If during ECMO weaning the cardiac output assessed with our method is not sufficient, further diagnosis should help clarifying the underlying pathophysiology. In case of weaning failure due to left and right ventricular dysfunction, valvular dysfunction, incomplete filling or insufficient venous return echocardiography as well as pulmonary catheterization should be considered^5^.

## Conclusions

This in-vitro study explored the relationships between blood gas content (CO_2_ and O_2_), blood flow and the elimination of these gases. We show that gas exchange during ECMO weaning might help in predicting the pulmonary blood flow. Our method could easily be transferred into a clinical setting, but would be limited if there are high shunts of blood in the lung or a high $\mathop {\text{V}}\limits^{.}$/${\dot{\text{Q}}}$ mismatch.
